# Analysis of small extracellular vesicles from dried blood spots

**DOI:** 10.3389/fmedt.2025.1494239

**Published:** 2025-01-27

**Authors:** Rikke Bæk, Jenni Kathrine Sloth, Mohammad Mehedi Hasan, Getnet Midekessa, Malene Møller Jørgensen

**Affiliations:** ^1^ Department of Clinical Immunology, Aalborg University Hospital, Aalborg, Denmark; ^2^ Research Department of Maternal and Fetal Medicine, Elizabeth Garrett Anderson Institute for Women’s Health, University College London, London, United Kingdom; ^3^ Department of Pathophysiology, Institute of Biomedicine and Translational Medicine, University of Tartu, Tartu, Estonia; ^4^ Institute of Veterinary Medicine and Animal Sciences, Estonian University of Life Sciences, Tartu, Estonia; ^5^ Department of Clinical Medicine, Aalborg University, Aalborg, Denmark

**Keywords:** small extracellular vesicles, dried blood spots, EV Array, phenotyping, whole blood

## Abstract

This protocol paper describes how to extract small extracellular vesicles (sEVs) from dried blood spots (DBS). The methodology is described in detail and offers further evidence that the extracted particles are sEVs using western blotting (anti-CD9, CD63 and CD81) and fluorescence nanoparticle tracking analysis (fNTA). In addition, we present evidence that approximately 40% of the sEVs were recovered from DBS compared with EVs analyzed from plasma directly. The protocol proves to be robust, reliable and displays very interesting performances even after several weeks (up to 3 weeks) of storage of the DBS when analyzing the sEVs using protein microarray for the presence of the markers CD9, CD63, CD81, EpCAM, Flotilin-1, CD62E/P, CD142 and CD235a. These findings have important implications for using sEVs as future potential diagnostic tools by supporting the validity of less-invasive methods that can be implemented within vulnerable populations or in the field.

## 1 Introduction

The term extracellular vesicles (EVs) refers to nanoscale particles that are comprised of a lipid bilayer membrane and contains variable cargo of DNA, RNA, and proteins. EVs can be isolated from all biological fluids; their presence reflecting a balance between secretion and uptake by the various local cell types. Exosomes are a class of EVs classically defined as small spherical EVs, 30–150 nm in size, and originating from the endosomal/multivesicular body system (1). This rigid definition is being called into question with the advance of research, which has come to view EVs as a continuum in terms of size, biogenesis, and molecular constitution (2). Research on EVs has seen an exponential increase in recent years, demonstrating an impressive variety of cargoes, including proteins and RNA species, many of which constitute potential biomarkers.

However, investigation of EV biology and clinical translation are not adequately supported by current manufacturing and characterization technologies (3). In particular, attribution of a purity grade and determination of the particle concentration of EV preparations in a reproducible and scalable/cost-effective fashion requires further improvement (4). Therefore, the direct use of circulating EVs for disease diagnosis has been limited by the current lack of methods to purify, measure, and characterize these. The protein composition of the EVs can be determined by a broad range of technologies as e.g., high sensitive flow cytometry (hFCM) (5), Luminex (6), SOMAscan (7), Simoa (8), Proximity Extension Assay (PEA) (9), proteomics and immunoblotting, as well as by array technologies using antigenic capturing of small EVs (sEVs) including exosomes by protein microarrays (10).

Venous blood is a conventional source of circulating EVs, which requires blood sampling by authorized personnel and immediate isolation of the plasma/serum containing the vesicles. Blood collected and dried on a paper card, dried blood spot (DBS), are of growing interest as a sampling method that can be performed outside care facilities by capillary puncture and afterwards transported in a simple and safe manner by mail. The idea of using blood collected on a paper card made of cellulose is ascribed to Ivar Christian Bang (1869–1918), the father of modern clinical microanalysis (11). In 1913, Bang determined glucose from eluates of DBS and, later, also performed nitrogen measurements using the Kjeldahl method with this filter paper technique. Subsequently, several investigators reported the use of DBS for serological testing to diagnose syphilis. This paper shows that intact sEVs can in fact be obtained from DBS, and that intact sEVs can be detected in extracts from DBS even after prolonged storage.

DBS is increasingly being used for a variety of tests, including those for infectious diseases [e.g., HIV, hepatitis (12)], cardiovascular diseases (13), metabolic conditions (14), and pharmacokinetic monitoring (15). Its versatility makes it a valuable tool in both routine screening and chronic disease management, reducing the need for multiple in-person visits to hospitals. Several methods for EV protein characterization exist; however, it is challenging to choose the most optimal one as reviewed by Ramirez and co-workers (16). The main issue is that many methods are limited by sample purification, labeling, and selection of optimal combinations of biomarkers. With the use of protein microarray technology, the EV Array made it possible to semi-quantify and phenotype sEVs directly from cell cultures as well as from plasma and serum. In perspective, the EV Array technology has made it possible to setup large-scale experiments monitoring effects of various cell stimulations and conditions on the production of sEVs. Even more importantly, this was performed without any time-consuming isolation or enrichment of the sEVs prior to analysis.

Preanalytical variations are one of the biggest hurdles in EV diagnostic translation and using DBS could circumvent some of these variations as they are stable at room temperature for extended periods, allowing for easier and cheaper transportation and storage without the need for refrigeration or immediate processing. This stability also makes it easier for clinics or health programs to collect and store samples for later analysis, even when logistical challenges are present.

Here, we present a step-by-step protocol to extract sEVs from DBS for further characterization directly by microarray capturing (EV Array). More information about the advantages and limitations with respect to the current method can be found in the filed patent (US patent no. US12,055,537).

## 2 Materials and methods

### 2.1 Venous whole blood collection

Whole blood was collected through venous draw into EDTA K3, CPDA or Serum Clot activator 6 ml Vacuette™ tubes (Greiner Bio-one GmbH, Austria). EDTA K3 and CPDA was subsequent a 30 min incubation centrifuged at 1,500 × g for 6 min and plasma was isolated and stored in aliquots at – 40°C.

### 2.2 Dried blood spot collection

Blood spots were either collected directly from serum tubes (described below) or from fingertip prick on “whole cells blood card” (ArrayIt Incorporation, CA, USA). The cards were left to dry for 1 h before being placed in a sealable bag and stored at room temperature for up to 3 weeks. Supplementary Material S1 describe a detailed protocol for the fingertip prick collection.

Serum tubes was immediately after blood draw inverted 5 times and volumes of 50 µl whole blood was pipetted onto the blood cards within a few minutes. The cards were left to dry for 1 h before being placed in a sealable bag and stored at room temperature for up to 3 weeks.

### 2.3 Extraction of EVs from dried whole blood spots

**Figure 1:**
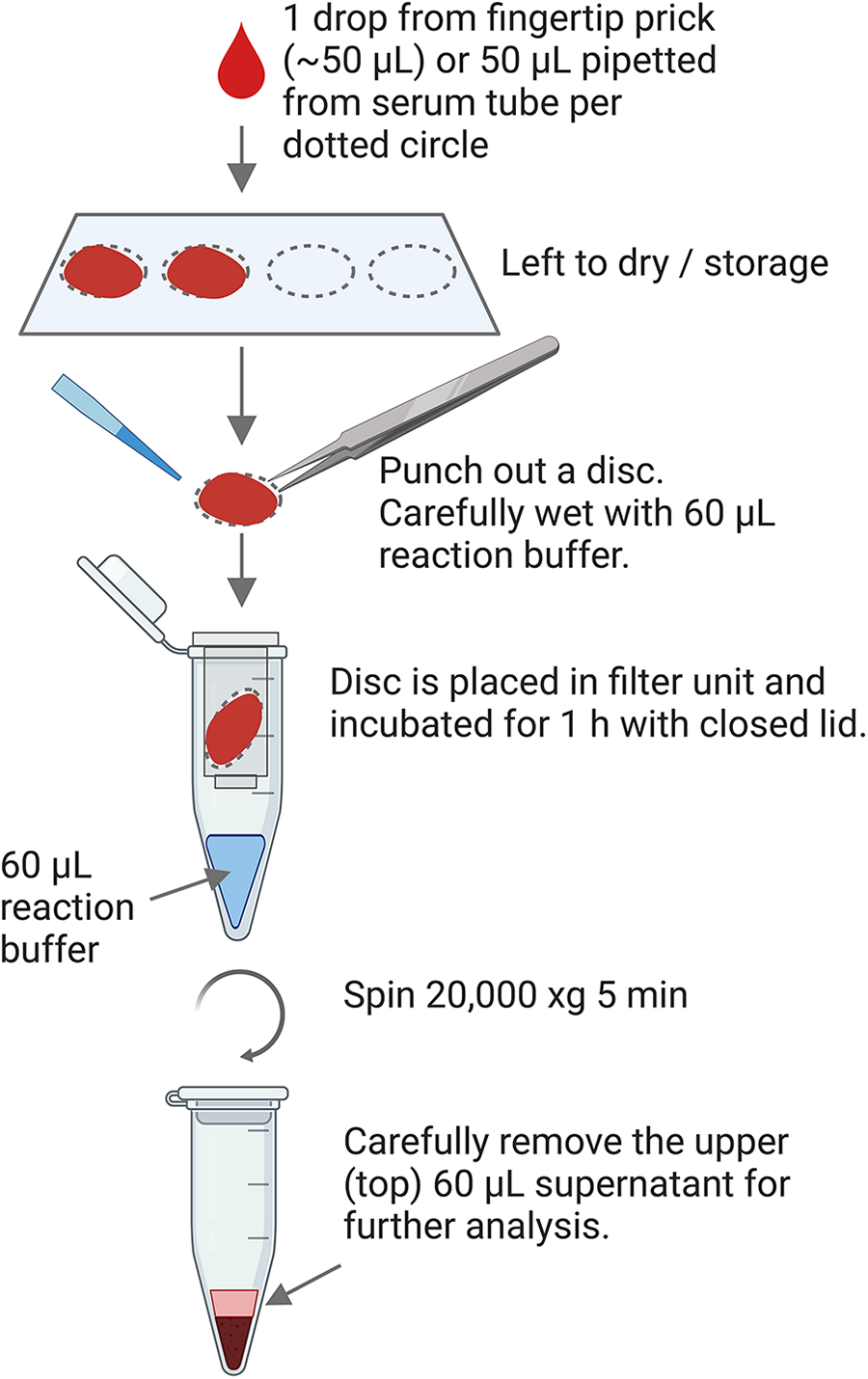
Graphical workflow of the extraction procedure from DBS.

Extraction of EVs from the DBS starts with excising a circular disc from the sample card (12.5 mm diameter) using a disc punch designed for the purpose. The excised disc is wetted with 60 µl of Reaction Buffer (PBS, 0.05% Tween20®) and placed in a spin column, which is put into a collection tube containing additional 60 µl Reaction Buffer. The spin column is closed, and the assembly is incubated for 1 h at room temperature, following a centrifugation at 20,000 × g for 5 min. Afterwards, the spin column and excised circle is discarded, and 60 µl sample comprising sEVs are transferred from the top of the supernatant to a new sample tube. Figure 1 illustrates the extraction procedure and Supplementary Material S1 describe a detailed protocol for the extraction.

### 2.4 EV Array

During production of the microarray, antibodies were printed on epoxy-coated slides (75.6–25.0 mm; SCHOTT Nexterion, DE) using either a sciFLEXARRAYER S12 installed with a PDC60 with coating type 3 (Scienion AG, DE) or a SpotBot Extreme Protein Edition Microarray Printer using a 946MP4 pin (ArrayIt Corporation, CA, USA). Biotinylated human immunoglobulin G (100 μg/ml) was used as positive control and phosphate-buffered saline (PBS) with 5% glucose was used as negative control. After the printing procedure, the slides were left to dry at room temperature overnight before further analysis.

Eight anti-human antibodies were used (with the corresponding clone, if available): CD9 (SN4/C3-3A2), CD81 (1.3.3.22, LifeSpan BioSciences, WA, USA), CD63 (MEM-259, Biolegend, CA, USA), Flotillin-1 (Abcam, MA, USA), EpCAM (0.N.277, Santa Cruz Biotechnologies, CA, USA), CD62E/P (BBIG-E(13D5), CD142 (323514), CD235a (R10, R&D System, MN, USA). All antibodies were diluted in PBS with 5% glucose and printed in triplicates at 200 μg/ml.

The EV Array analysis was performed as described by Jørgensen et al. (17). In short, the microarray slides were blocked (50 mM ethanolamine, 100 mM Tris, and 0.1% sodium dodecyl sulfate, pH 9.0) before incubation with sample (10 µl of plasma or 50 µl of DBS extracted EVs) diluted in wash buffer (0.05% Tween20® in PBS). The microarray slides were incubated in Multi-Well Hybridization Cassettes (ArrayIt Corporation, CA, USA) at room temperature for 2 h followed by overnight incubation at 4°C. After a wash, the slides were incubated with biotinylated detection antibodies (anti-human CD9 (SN4/C3-3A2), anti-human CD63 (AHN16.1/46-4-5), and anti-human CD81 (1.3.3.22), LifeSpan BioSciences, WA, USA) diluted 1:1,500 in wash buffer for 2 h. After additional washing, incubation for 30 min with cyanine 5–labeled streptavidin (Life Technologies, CA, USA) diluted 1:1,500 in wash buffer was carried out for detection. Before scanning, the slides were washed in wash buffer followed by MilliQ water and dried using a Microarray High-Speed Centrifuge (ArrayIt Corporation, CA, USA). Scanning and spot detection were performed as previously described (17).

### 2.5 Isolation of EVs for western blotting

One ml of plasma (diluted 1:1 with PBS) or DBS extracted material (from 40 spots) were centrifuged at 13,200 × g for 22 min. Subsequently, the supernatant was filtered (pore size 0.2 µm) to remove large particles. Filtered supernatants were then ultracentrifuged at 100,000 × g for 16 h and pellet washed in PBS before ultracentrifugation at 100,000 × g for 2 h.

### 2.6 Western blot

After ultracentrifugation of plasma or extracted DBS material, the pellets were solubilized in Ripa buffer (50 µl). The samples were preheated at 70°C for 10 min. SDS/PAGE was carried out on a 12% Bis/Tris protein gel (NuPAGE, ThermoFisher Scientific, MA, USA), and proteins were transferred to IBlot™ membranes (ThermoFisher Scientific, MA, USA). The membranes were blocked in iBind™ Blocking solution and probed with anti-human CD9 (SN4/C3-3A2), CD81 (1.3.3.22, LifeSpan BioSciences, WA, USA), or CD63 (MEM-259, Biolegend, CA, USA) antibody (LifeSpan BioSciences, WA, USA) and secondary antibody (anti-mouse HRP, ThermoFisher Scientific, MA, USA) using the iBind™ Western System and iBind™ solution kit (ThermoFisher Scientific, MA, USA). For visualization, blots were exposed to Pierce® Enhanced ECL West Pico PLUS Substrate and measured by C-DIGIT blot scanner (LI-COR Biosciences GmbH, DE).

### 2.7 Size exclusion chromatography (SEC) purification of EVs

Isolation and purification of EVs from plasma or DBS were performed following the earlier methods (18). In brief, the commercially available size exclusion chromatography (SEC) column (qEVsingle/70 nm by Izon Sciences, UK) was used to isolate EVs. Before isolation of EVs, the column was prewashed using 10 ml filtered (0.2 µm Minisart® syringe filters, Sartorius, DE) Dulbecco's phosphate-buffered saline (DPBS, Sigma® Life Science, UK). Afterwards, the 150 µl sample (plasma or DBS extracted EVs) was layered on the top of the column filter and allowed to pass down the filter. DPBS was immediately added on the top of the column filter. A total of 20 fractions were collected separately (each 200 µl). To identify the EV fractions with high concentration and low protein contamination, all the samples were analyzed using ZetaView® NTA PMX 120 V4.1 (Particle Metrix GmbH, DE). According to the manufacturer's protocol, the protein concentration of each fraction was measured with the Quick Start™ Bradford Protein Assay (Bio-Rad, CA, USA). The EV fractions (5–9) for each sample were pooled, concentrated, and used for labeling and NTA analysis.

### 2.8 EV labeling with lipophilic membrane dye (CMG)

EV labeling with lipophilic membrane dye was performed based on prior published method (19). EVs purified by SEC were diluted in PBS to a particle concentration of ∼1 × 10^10^ particles/ml. Prior to incubating EVs with CellMask™ Green plasma membrane stain (CMG, Thermo Fisher Scientific, MA, USA), 1 µl of CMG dye with a stock concentration of 5 mg/ml was diluted in 50 µl PBS. Later, 1 µl of the diluted CMG dye was added to 9 µl diluted EVs and incubated at room temperature for 1 h in the dark. Subsequently, the incubated samples were added to 990 µl of PBS.

### 2.9 Nanoparticle tracking analysis of EVs

Nanoparticle tracking analysis (NTA) was conducted using a ZetaView PMX 120 V4.1 instrument (Particle Metrix GmbH, DE). NTA's laser and microscope were autoaligned using a known concentration of both 100 nm polystyrene and fluorescent Yellow-Green standard beads (Applied Microspheres B.V., NL). The standards and EV samples were diluted in particle-free water and PBS for analyses, respectively (20). For scatter mode, the particle number and size distribution were measured at 11 frames per cycle with a sensitivity of 72 and a shutter value of 100. The size and concentration of fluorescently labeled EVs were measured at a sensitivity value set at 90 and the ZetaView® software was used to collect and analyze the data.

### 2.10 Statistical analyses

Statistical analyses were performed using GraphPad Prism v8.4.2. Pearson correlations were conducted to assess the concordance of sEVs ratios across venous blood and DBS. The comparison between the concentration of CMG-labeled EVs at the scatter and fluorescent modes of NTA was assessed using the two-tailed Student *t*-test. Data are presented/displayed as mean ± SD (*n* = 3).

## 3 Results

The procedure to extract sEVs from DBS is illustrated in Figure 1. The first step is to punch out the circles, and after the extraction procedure the end product is a 2-phase separation (not a pellet) from which the sEVs can be recovered from the upper phase. From a DBS originating from ∼50 µl finger prick blood (or 50 µl non-coagulated venous blood), the sEVs are recovered in 60 µl of buffer. The extract is slightly red, as extensive hemolysis has occurred during the drying and extraction procedure.

### 3.1 Extraction buffers and filter units

During the development of the extraction protocol, several types of filter paper were tested alongside with different compositions of buffers [PBS with various amounts of detergent and commercial buffers (content unknown)] and timings of the extraction step (examples are shown in Supplementary Material S2). The conclusion of these preliminary investigations was that the difference was minimal and therefore it was decided to use PBS with 0.05% Tween20®, as this buffer is optimized as incubation and wash buffer for the EV Array analysis. However, if other types of methods should be used downstream of the extraction for EV characterization, the extraction and reaction buffers can easily be exchanged, although it will probably affect the extraction efficiency. During the preliminary investigations it was found that addition of detergents was not crucial for the extraction (Supplementary Material S2), however if detergent is added it was found urgent to keep the concentration below the critical micelle concentration (CMC) of the detergent.

Furthermore, several filter units with different pore size and/or molecular weight cut-off (MWCO) was tested in combination with several settings of centrifugation (times and g-forces) (data not shown). All filter units with a MWCO were found to cloth at some degree, so the best results were gained with a filter unit which physically holds back the excited filter disc allowing all eluted content to pass (data not shown).

### 3.2 EV characterization

**Figure 2:**
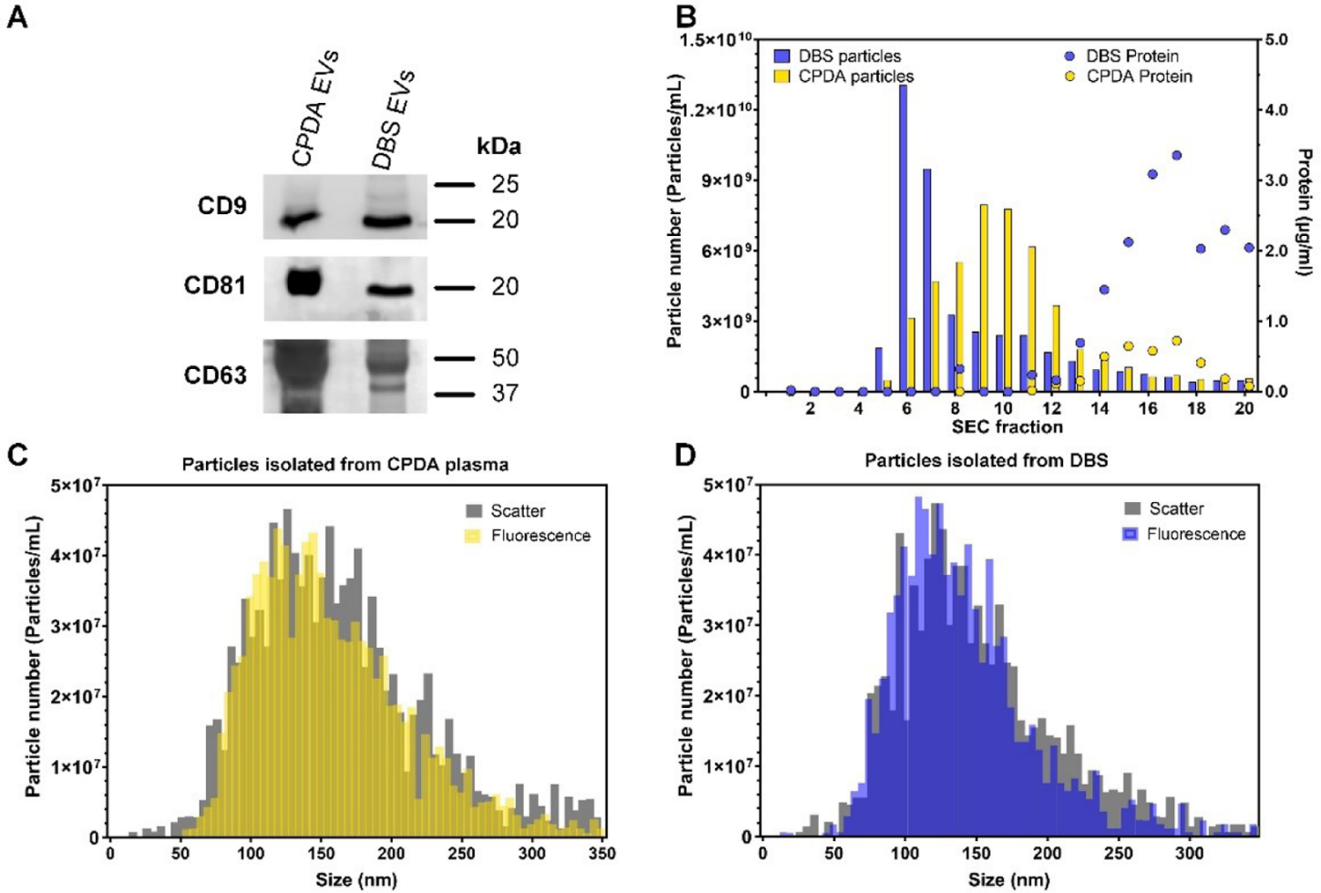
Characterization of EVs enriched from CPDA plasma or EVs extracted from DBS. **(A)** Western blotting of CD9, CD81 and CD63 for EVs enriched by ultracentrifugation. **(B)** Nanoparticle tracking analysis (NTA) and protein measurements of fractions obtained from SEC purification. **(C,D)** Scatter and fluorescent NTA of SEC fraction pool (5-9) labelled with CMG membrane dye.

To be able to perform detailed EV characterizations, further enrichment and isolation procedures were needed. To perform a western blot, EVs extracted from 40 DBS was pelleted by ultracentrifugation and the extraction from DBS showed comparable bands for CD9, CD81 and CD63 as EVs pelleted from venous CPDA plasma (Figure 2A).

Both CPDA plasma and the EV extraction from the DBS contain many types of particles e.g., lipoproteins, cell remnants and protein aggregates. To verify the contents of EVs, a SEC purification was performed from EVs extracted from 8 DBS.

The particle and protein concentrations were measured for each fraction and the EV containing fractions (5–9) were pooled (Figure 2B). The nanoparticle tracking analysis (NTA) measured both in scatter and fluorescent mode (CellMask™ Green, membrane stain) revealed that there is similarity between the modes of EV size obtained from different sample types ranging from 110 to 135 nm, with an average mode size of 130.5 ± 6.2 nm for CPDA and 115 ± 4.8 nm for DBS samples.

### 3.3 Reproducibility and storage

The reproducibility of the extraction methods was further investigated using EV Array (10, 17), a microarray-based multimarker method with high sensitivity, that allowed us to characterize sEVs from each individual DBS. A small panel of markers was chosen for capturing the sEVs including CD9, CD63, CD81, Flotilin-1, EpCAM, CD62E/P, CD142 and CD235a. For each sample, every marker was analyzed in triplicates.

**Figure 3:**
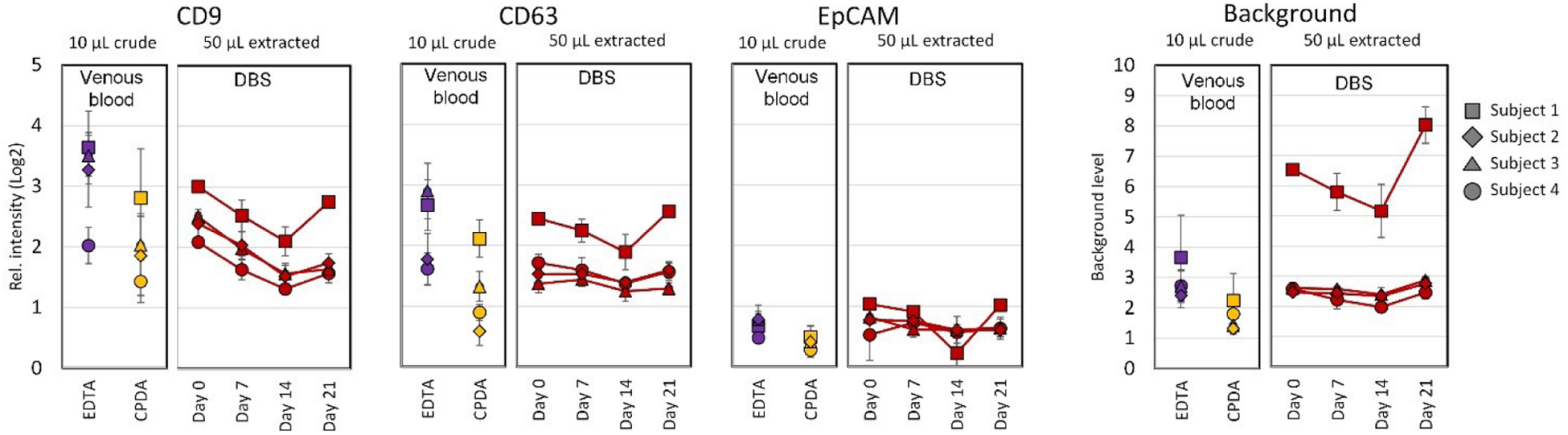
Result from EV Array analysis for the markers CD9, CD63 and EpCAM from either venous plasma (EDTA or CPDA) or DBS extracted EVs. From four subjects 50 µl serum blood was depositioned on the spots in 3 repetitions. sEVs was extracted either at day 0 or after storage for 7, 14 or 21 days. The level of background is given for all measurement (the far right). Error bars indicate SEM.

For the EV Array analysis, each sample was loaded into a well and the outcome intensity was calculated relatively to the background for that sample. This is important to notice, as each sample of biological origin has its own reaction with the surface and components of the microarray. Even plasma samples from venous blood from different persons will have different backgrounds even if they have been through the same pre-analytical treatments. Most of the times the background will increase with increasing amount of sample loaded, so it is a balance to find the most optimal signal-to-background sample amount. The most optimal results were found when loading 50 µl of extracted EVs, which in comparison gave similar results as when loading 10 µl of venous plasma (Figure 3). It is estimated that the DBS extraction from one drop of blood (∼50 µl) contains a similar amount of intact sEVs as 20 µl of venous blood processed to plasma, which gives a recovery of ∼40% of the sEVs.

To investigate the variation and sensitivity of the extraction process before and after storage, an experiment were performed where precisely 50 µl of serum blood was depositioned by pipetting onto the filter papers (18 spots per subject). The DBS was stored for up to 21 days and the results revealed a robust elution procedure (Figure 3).

On average, the 3 repeated extractions followed by EV Array analysis gave a coefficient of variance on Day 0: 5.8%; Day 7: 9.2%; Day 14: 9.1%; and Day 21: 6.8%.

As described earlier, the background for each EV Array measurement varies and particular one of the subjects (Figure 3, subject 1) had a higher level of background, which increased particularly after 21 days of storage. This sample was also found to have a more deep red color, indicating a higher degree of hemolysis after the storage. The hemolysis was not found to be associated with an increase in red blood cell-derived EVs as seen by the marker CD235a (Supplementary Material).

### 3.4 Quantitative vs. qualitative results

**Figure 4:**
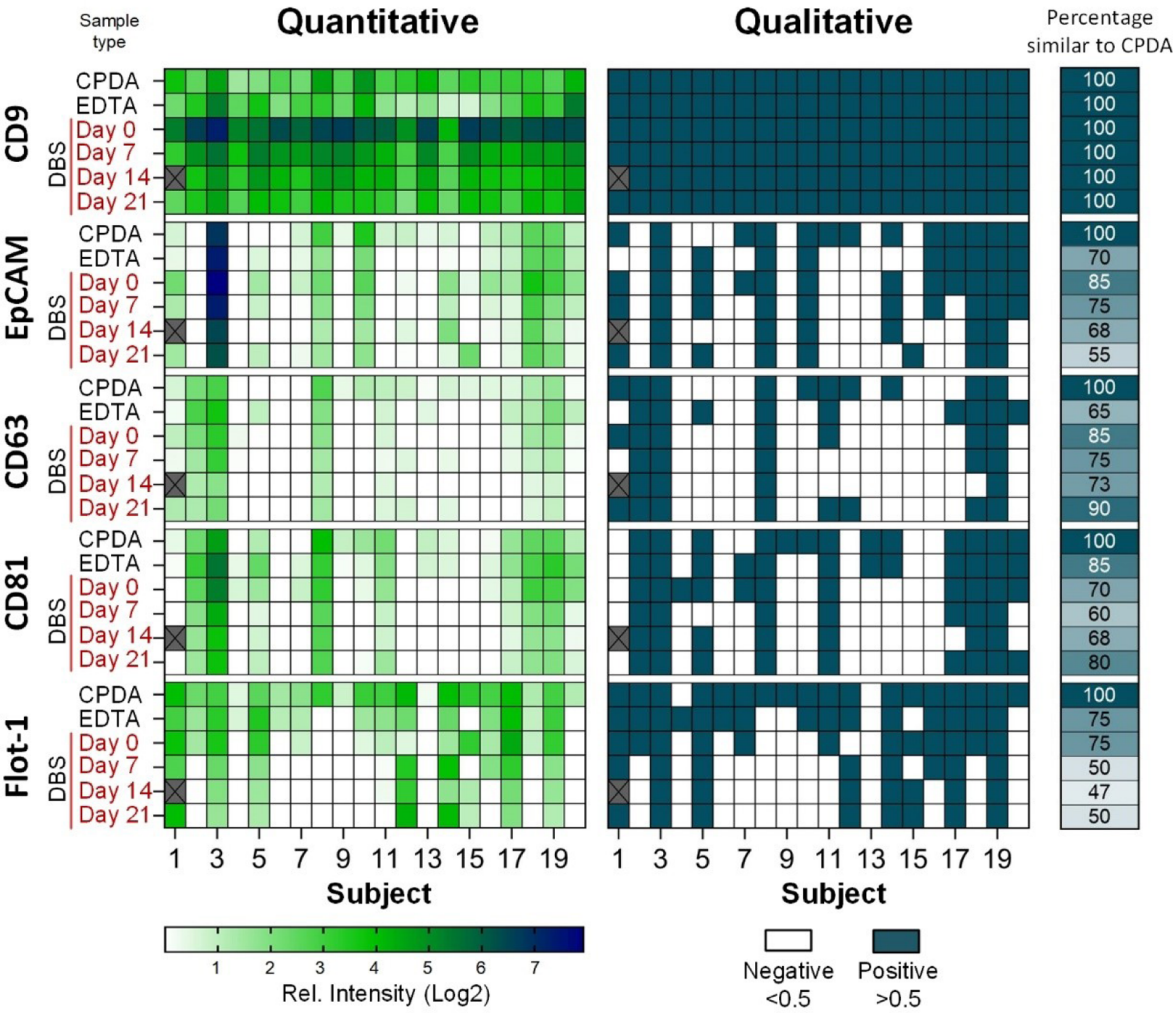
Heatmaps of the results from EV Array analysis with the markers CD9, EpCAM, CD63, CD81 and flotilin-1 used for capturing. sEVs extracted at day 0, 7, 14 and 21 from DBS from 20 subjects from finger prick and compared to plasma isolated from venous blood (CPDA and EDTA). Left side: Intensities relative to background (Log2); Right side: Qualitative measure using a threshold of 0.5 of relative intensity. The percentage of positive or negative signals similar to CPDA is given next to the heatmap (far right side).

The above tests showed a high reproducibility for the extraction of sEVs from DBS even after storage. However, this was only possible to test, as precisely 50 µl of whole blood was depositioned by pipetting. In order to demonstrate the variance of sample loading and storage, 20 healthy subjects were asked to fill 1 card with 4 circles by finger prick. The DBS was stored at room temperature and EVs were extracted after 0, 7, 14 and 21 days and analyzed by the EV Array. The results are shown as heatmaps in Figure 4.

The results obtained from the 20 subjects showed a natural occuring biological variation between the subjects. It is also observed, that the type of collection tube (CPDA or EDTA) for the venous blood has an impact of the EVs characterized, which confirms the observations made previously when studying the impact of pre-analytical treatments (21).

There are some discrepancies between results obtained from sEVs extracted from DBS both compared to the type of venous blood isolated plasma, but also during storage. The main cause for these discrepancies is probably due to the differences in loading of the blood from a finger prick. It was observed, that for the 20 subjects, the amount of blood placed within the paper circles varied a lot. They got instructions to fill the circles, however, some had a better penetration of the skin than others and therefore were able to soak the circles entirely. Others had a lower blood flow and only just “colored” the circles with blood. Therefore, it was not possible to obtain quantitative results from the EV Array analysis using DBS, therefore, the data was transformed to qualitative results using a treshold of 0.5 in relative intensities (Figure 4, right side).

With CPDA as reference it was possible to detect the highly expressed marker CD9 for all donors for up to 21 days of storage.

## 4 Discussion

Obtaining enough EVs for robust protein characterization often requires large sample volumes, which can be impractical or difficult. To perform the western blot analysis in this study, material from 40 DBS was used to be able to visualize the presence of CD9, CD63 and CD81. Prior to the analysis isolation/enrichment of the EVs was performed by ultracentrifugation and most methods for characterization of EVs requires a pre-isolation/purification step. However, the EV Array analysis requires only small amounts of sample material (one DBS) and can therefore be performed directly on eluate from a single DBS.

DBS collection from finger prick is minimally invasive, inexpensive, and robust to long-term storage (22). This study show that finger prick DBS can be implemented effectively to measure sEVs and are therefore a viable alternative to invasive venous blood draws. Our findings solidify the case for using finger prick DBS in research of sEVs, which could open up for using DBS in national screening programs for e.g., diabetes or cancers. This, however, requires identification of robust EV biomarkers in additional biomarker discovery studies. In this study, we collected specimens from a relatively homogenous cohort of healthy adults, which limits our ability to assess how clinical conditions or other individual differences might affect the sampling and subsequent analysis.

The main disadvantage of the DBS card method is the quantity of sEVs recovered compared to the yields obtained from fresh venous blood samples. For the characterization of sEVs using the EV Array, the obtained amount of sEVs from one DBS was shown to be applicable. However, this will not be the case for other types of analysis such as flow cytometry and WB, due to the low amount/numbers of sEVs extracted from the DBS. However, other ultrasensitive analytical technologies that span a wide dynamic range can probably also be used to detect protein in low abundance subpopulations or low sEV amount material. These methods include Luminex (6), SOMAscan (7), Simoa (8), MesoScale Discovery (MSD) (23), Droplet-based Extracellular Vesicle Analysis (DEVA) (24), and Proximity Extension Assay (PEA) (9). These techniques all have their advantages and disadvantages as reviewed by Shami-Shah et al. (25) e.g., whether a further purification of the sEVs is needed or whether the high degree of hemolysis will be a hindrance for the analyses.

The above-mentioned techniques all target the protein content of the EVs, however several studies have found the nucleotide content (coding and noncoding RNA and DNA) good as biomarkers as well, as reviewed by Momen-Heravi et al. (26). The technologies of nucleotide detection and sequencing is developing fast gaining new high-sensitive methods. The main advantages of using nucleotides as biomarker is the possibility to enhance the signals by PCR prior to detection.

The detection of EV markers through WB and fluorescence NTA was used to confirm the presence of EVs in the solutions obtained at the end of the isolation protocols. The objective was not to perform a detailed characterization of the markers, but to look for differences in their detection between the different methods of blood drawing (Figure 2).

Venous blood collected on blotting paper is an alternative method of sampling with many advantages compared to serum or plasma specimens. The lower analytical sensitivity of assays performed on DBS compared to serum/plasma is one of the limitations of DBS, since biomarkers can be present at very low concentrations during different diseases and stages of disease. The data from this study suggest that the analytical sensitivity of DBS in combination with EV Array analysis is sufficient to detect EV markers even at low levels. However, additional optimization will probably be needed dependent on the molecular characterization of the marker.

The size of the skin penetration depends on the size and type of lancet and influences the amount of blood that can be collected. Standard DBS cards use pre-printed circles of 12 mm in diameter to receive between 50 and 70 µl of blood (11, 27). The massage of the finger before puncture and warming of the hands in warm water can facilitate the sampling. After puncturing it is important to exert a strong intermittent pressure to maintain the bleeding and complete the blotting paper card. Characterizing sEVs from 20 healthy subjects revealed biological variation between subjects as expected. However, the experiments also showed a great variation and fluctuation of sample volume loaded within each subject. The single most important factor for minimizing “fluctuations” of the sample volume is undoubtedly a correct technique of capillary blood collection, which can only be achieved by careful instructions and experience.

In 2018, Velghe and Stove presented the Capitainer-B device (commercialized by Capitainer AB, Stockholm, Sweden) (28). The device is equipped with an inlet port to which a drop of blood (e.g., obtained via a finger prick) is introduced, resulting in the filling of a capillary microchannel with a fixed volume. Upon filling this capillary channel completely, a thin film at the inlet dissolves, resulting in the absorption of the excessive amount of blood by a paper matrix, leading to the separation of the excess blood and the filled channel. Finally, upon dissolving of a thin film at the outlet, the capillary channel is emptied through capillary forces, resulting in the absorption of the blood by a pre-perforated paper disc (29).

The importance of studying EVs in a hospital setting to complement the diagnosis and prognosis of several diseases has been well demonstrated using the EV Array technology (30–33). Nonetheless, we believe that the workflows from the collection of samples aimed at the isolation, processing, and characterization of EVs to yield significant results to be applied on patients need to be standardized. Specifically, the different isolation methods can yield different types of EVs and, thus, omics studies performed on them could give incomparable results. Besides, not all methods are applicable in a hospital setting.

The COVID-19 global pandemic influenced significant changes to the way patients are monitored, particularly those with chronic disease or those who are immunocompromised, and the need to minimize visits in hospitals and clinics, combined with new tests/therapies to be monitored has accelerated the need of these volumetric blood collection devices (34). DBS is a clinically relevant tool for decentralized sampling. DBS can contribute more broadly to improve access to *in vitro* diagnosis,.

DBS is an excellent tool for longitudinal monitoring to follow treatment progress or screen for relapse in various medical conditions. Regular and frequent testing (e.g., daily, weekly, or monthly) becomes feasible, even in situations where it may not be practical to visit a healthcare provider frequently.

Sampling on DBS can be performed in locations with limited healthcare infrastructure, which eliminates many of these costs by allowing for remote sample collection. It also reduces the logistical burden associated with traveling to appointments, making it more affordable for both patients and healthcare systems. This is particularly advantageous for underserved populations living in rural, remote, or impoverished areas where healthcare facilities are scarce or far away (35).

DBS simplifies the process of sample collection, reducing the time spent in waiting rooms or undergoing more invasive procedures. The ability to self-collect blood samples quickly and easily at home means patients can save time, which is particularly beneficial for busy individuals or those with mobility challenges. Sampling on DBS typically involves a small prick to the finger, ear or heel, which is much less invasive than traditional blood draws that require a needle inserted into a vein. This can be especially important for children or elderly individuals who may be more sensitive to pain or who have difficulty tolerating conventional blood draws (35).

As shown in this study, DBS are stable at room temperature for extended periods, allowing for easier transportation and storage without the need for refrigeration or immediate processing. In underserved areas with limited access to cold storage or where samples need to be transported over long distances, this is a significant advantage. This stability also makes it easier for clinics or health programs to collect and store samples for later analysis, even when logistical challenges are present.

Diagnostic tests on DBS are consequently difficult to integrate into the laboratory workflow. Hence, the tests on DBS require rigorous validation in clinical laboratories to guarantee the quality of the results. The pre-analytical steps of laboratory analyses performed on DBS keep a manual character, however, automated systems for handling DBS in a clinical setting do exit as tested by Carling et al. (36).

Traditional blood draws often require a larger volume of blood, which can be harder to obtain from infants, young children, or frail elderly patients. DBS, on the other hand, requires only a tiny amount of blood, which is less invasive and more suitable for those who may have difficulty donating larger quantities of blood. Since DBS requires only a small puncture of the skin, the risk of infection is lower compared to traditional venipuncture (inserting a needle into a vein), which may cause more complications, especially in individuals with fragile veins or weakened immune systems (37).

The limitations of this study include the sampling of a predominantly population with a high socioeconomic status. Future research with a more diverse sampling is needed for producing more generalized findings. We recruited people that was already involved in research, thereby lending the sample to bias. For this study, we were unable to request at-home DBS collection. A follow-up study including actual at-home DBS collection and retention rates would strengthen the conclusions of this preliminary assessment.

A widespread concern in the biomedical research community is the gap between the basic research carried out in the laboratories and the clinical setting where the new biological information should have an impact. Many researchers have directed their efforts towards bridging that gap and look for ways to translate lab findings into clinical solutions, therefore emerging the translational research. The translational research on EVs is not foreign to this goal: the current knowledge about EVs, mostly developed *in vitro*, has been proposed to be applied in a daily hospital routine giving answers to specific health queries (38). The possible applications ranges from diagnostic to therapeutic objectives, including disease monitoring and the search of prognostic biomarkers, among others.

## Data Availability

The raw data supporting the conclusions of this article will be made available by the authors, without undue reservation.
