# Supplementary material for: Analysis of small extracellular vesicles from dried blood spots
Source: Front Med Technol. 2025 Jan 27;7:1494239. doi: 10.3389/fmedt.2025.1494239 (PMC11808138; doi:10.3389/fmedt.2025.1494239)

Supplementary Material

1 S1: Detailed protocol

1. Wash hands for 30 sec with soap and warm water. Rinse hands thoroughly for 30 sec with warm water and dry completely. Decontaminate the finger with an ethanol swap. Place the lancet on the finger gently on the top of the lancet. Press down firmly on the lancet to pierce the fingertip.

2. Massage whole blood droplets out of the fingertip by applying firm pressure to the finger. Repeated firm pressure strokes from the base to the tip of the finger will accelerate blood flow. Saturate each 12.5 mm dotted circle completely so that the blood migrates outward and slightly beyond each dotted circle. Typically, 2-3 drops of whole blood placed in the center of the dotted circle are sufficient to saturate each 12.5 mm collection area. A slight excess of whole blood is preferable because the 12.5 mm disc punch excises precise 12.5 mm disc regions, allowing well-calibrated sample collection volumes if each 12.5 mm dotted circle is wetted completely.

3. After filling each of the four 12.5 mm dotted circle collection regions with whole blood, fold the top flap of the card inside the bottom flap and allow the collected blood spots to dry for 60 min at room temperature. The drying process removes water from the collected samples and stabilizes the collected samples. After the 60 min drying period, progress immediately to step 4. Alternatively, the cards can be placed inside a sealed plastic bag and stored at room temperature for future use.

4. The dried whole blood spots can be excised using a disc punch; for these experiments a 12.5 mm disc punch was used. Align the punch with the dotted circle at each collection location and press the disc punch firmly to excise the collection matrix containing the whole blood sample. For the EV Array analysis only a single 12.5 mm disc is required, which allows the remaining three discs to be saved and stored for future use. The disc punch should be rinsed with water and dried between each card to prevent sample cross contamination.

5. After the 12.5 mm disc containing the whole blood sample is excised with the disc punch, pick up the disc with fine-tipped forceps and wet each side of the 12.5 mm disc with 60 µl of reaction buffer (PBS with 0.05% Tween20®).

6. Apply additional 60 µl of reaction buffer into a collection tube and place a filter unit/spin column on top of the tube. Using fine-tipped forceps place the wetted disc into the filter unit orifice. Close the lid on the collection tube and incubate for 1 h at room temperature.

7. Place the filter unit-collection tube assembly in a high-speed microfuge and centrifuge at 20,000 x g for 5 min to elute the whole blood components and EVs off the collection disc and into the 60 µl of reaction buffer at the bottom. A properly eluted sample will appear as two layers in the bottom of the tube.

8. Harvest the eluted EVs in the top layer (approximately 60 µl) and transfer to another tube for either further analysis or storage (-80 °C). Make sure not touch the bottom layer as the sample then will be contaminated with cell remnants.

2 S2: Results from buffer optimization experiment

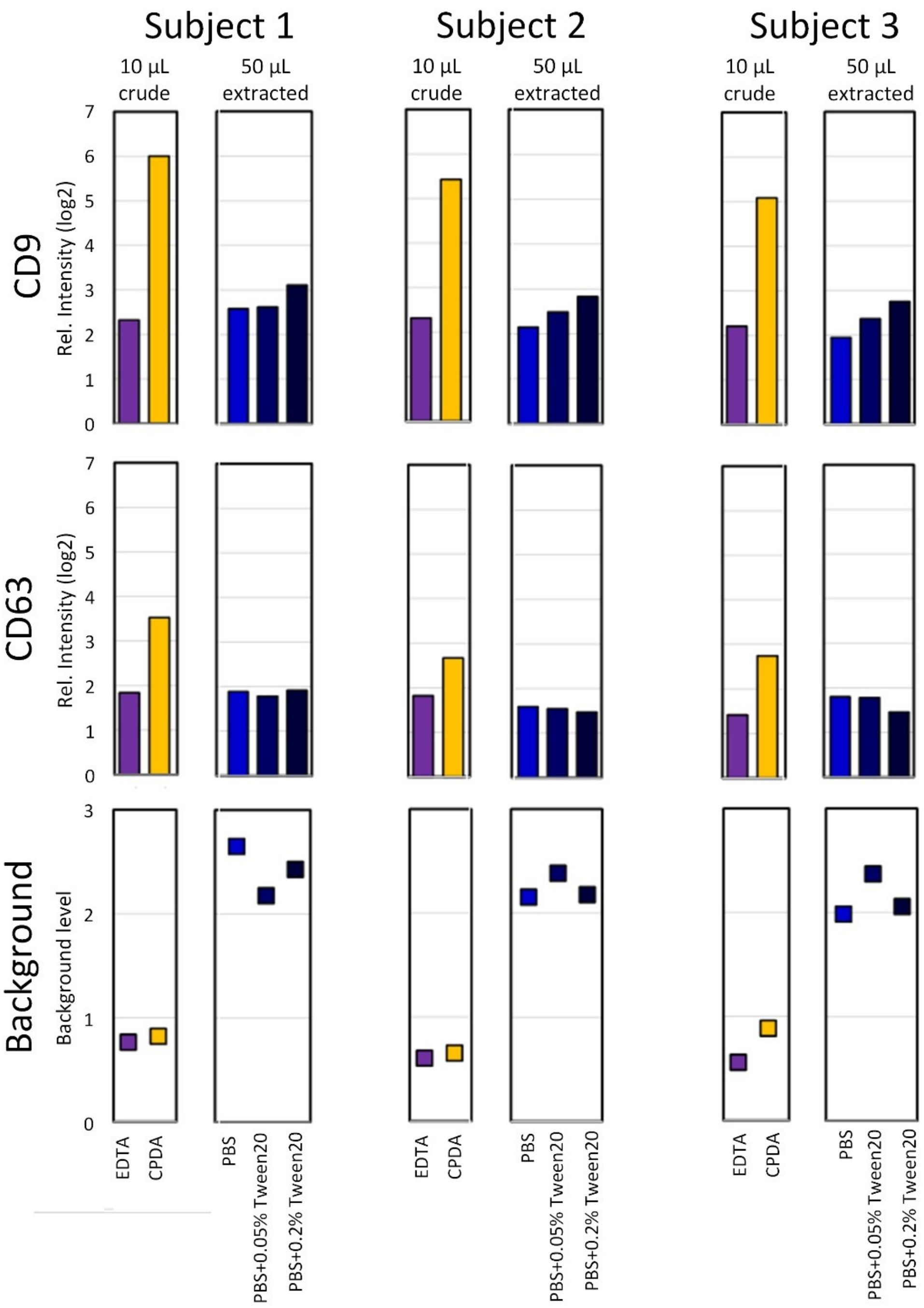

3 S3: Results from additional markers:

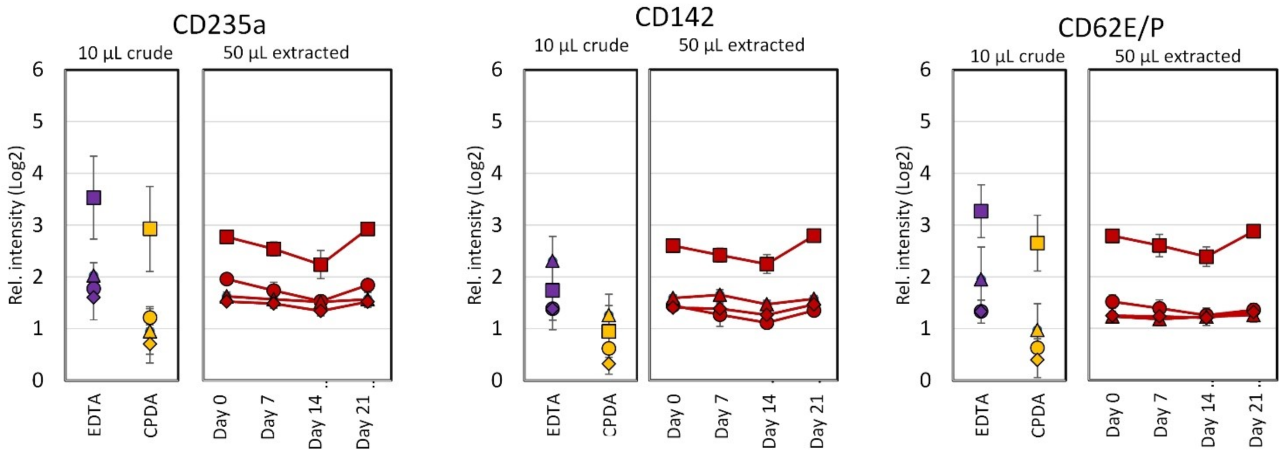

Supplement: Supplementary file 1 [file Datasheet1.pdf]
